# Overexpression of LcMYB90 Transcription Factor Enhances Drought and Salt Tolerance in Blue Honeysuckle (*Lonicera caerulea* L.) and Tobacco (*Nicotiana tabacum* L.)

**DOI:** 10.3390/ijms26073124

**Published:** 2025-03-28

**Authors:** Jing Chen, Chunyang Bian, Chunlin Fu, Qian Zhang, Dong Qin, Wenjun Hao, Manman Guo, Junwei Huo, Jiangkuo Li, Huixin Gang

**Affiliations:** 1 Key Laboratory of Biology and Genetic Improvement of Horticultural Crops (Northeast Region), Ministry of Agriculture and Rural Affairs, College of Horticulture & Landscape Architecture, Northeast Agricultural University, Harbin 150030, China; chen50300@163.com (J.C.); liweihua1999@163.com (C.B.); fu971008@163.com (C.F.); 13355170145@163.com (Q.Z.); dongq9876@126.com (D.Q.); 18536494227@163.com (W.H.); 18239400597@163.com (M.G.); huojunwei@neau.edu.cn (J.H.); 2 National-Local Joint Engineering Research Center for Development and Utilization of Small Fruits in Cold Regions, Northeast Agricultural University, Harbin 150030, China; 3 Institute of Agricultural Products Preservation and Processing Technology, Tianjin Academy of Agricultural Sciences, Tianjin 300384, China; 4 Tianjin Key Laboratory of Postharvest Physiology and Storage of Agricultural Products, National Engineering and Technology Research Center for Preservation of Agricultural Products, Tianjin 300384, China

**Keywords:** *Lonicera caerulea* L., *LcMYB90*, drought stress, salt stress, transient transfection

## Abstract

The MYB family plays a vital role in regulating plant stress resistance. However, the MYB protein in blue honeysuckle remains largely unexplored. In this study, the *LcMYB90* gene from blue honeysuckle ‘Lanjingling’ was stably transformed into tobacco and transiently transformed into blue honeysuckle to characterize its function. Subcellular localization analysis revealed that the LcMYB90 protein is localized in the nucleus. Transgenic plants overexpressing *LcMYB90* exhibited enhanced growth performance and higher survival rates under drought and salt stress conditions. These plants also showed increased levels of proline and chlorophyll, along with elevated activities of catalase, peroxidase, and superoxide dismutase. Conversely, malondialdehyde content and relative conductivity were lower, indicating that *LcMYB90* enhances tolerance to drought and salt stress. Under salt treatment, genes induced by osmotic stress, such as *NHX1* (Na^+^/H^+^ antiporters 1) and *SOS1* (salt overly sensitive 1), as well as antioxidant defense system genes like *SOD* (superoxide dismutase) and *CAT1* (catalase 1), were more highly induced in overexpression lines compared to the wild type, supporting the hypothesis that *LcMYB90* promotes salt tolerance by enhancing osmotic stress resistance and antioxidant capacity. Simultaneously, the transcription levels of genes involved in the abscisic acid pathway, including *NCED1/2* (9-cis-epoxycarotenoid dioxygenase *1/2*, *PYL4/8* (pyrabactin resistance-Like 4/8), and *CBL1* (Calcineurin B-like protein 1), were increased under drought stress conditions in the overexpression lines. These results suggest that *LcMYB90* maintains cellular homeostasis by promoting the expression of stress-related genes and regulating osmotic and oxidative substances, thereby improving tolerance to drought and salt stress.

## 1. Introduction

Environmental factors, especially high salinity and drought stress, profoundly impact plant growth, development, and crop yield. Over time, plants have evolved complex defense mechanisms to cope with various extreme environmental conditions. When encountering external stress, specific genes are activated or inhibited to mitigate damage [1]. Transcription factors such as AP2/ERF, MYB, NAC, WRKY, bZIP, and bHLH are vital in plant stress responses [2].

The MYB gene family is among the largest groups of transcription factors in plants, regulating various physiological processes such as the cell cycle, primary and secondary metabolism, and reactions to environmental stress [3,4,5]. MYB transcription factors feature a conserved MYB domain at their N-terminus for DNA binding. This domain contains four imperfect repeats, each about 52 amino acids long [6]. The MYB family is divided into the following four subgroups according to the number of repeats: 1R-MYB, R2R3-MYB, R-MYB (R1R2R3-MYB), and 4R-MYB [7,8]. The R2R3-MYB subgroup is the largest, comprising 126 of the 198 MYB genes found in the Arabidopsis genome [9]. Numerous studies have examined R2R3-MYB genes, emphasizing their regulatory function in gene expression under diverse abiotic stress conditions in model organisms and major agricultural crops. For instance, the overexpression of *MYB25* in Arabidopsis modifies salt stress and abscisic acid (ABA) sensitivity, playing a crucial role in the plant’s stress response [10]. *MYB15* enhances the expression of genes involved in ABA biosynthesis, thereby improving salt tolerance and drought resistance [11].

Under drought stress, plants accumulate ABA, which triggers adaptive responses to enhance stress resistance [12]. This leads to rapid stomatal closure to reduce transpiration and prevent water loss, while also enhancing the activity of key antioxidant enzymes to scavenge reactive oxygen species (ROS) [13]. In the early stages of plant drought stress, ABA-independent processes play a role in sensing and signal transduction, activating regulatory systems to respond to water deficit stress, including osmotic, ROS, mechanical, and other stress signals [14]. These early stress signals then up-regulate ABA biosynthesis by inducing the 9-cis-epoxycarotenoid dioxygenase (NCED). Transformants of the *NCED* gene exhibit higher drought resistance [15]. The ABA receptor pyrabactin resistance-like (*PYL*) gene, located upstream in the ABA signaling pathway, plays a crucial role in plant drought resistance by regulating the transcription of *PP2C* (protein phosphatase 2C), *SnRK* (Sucrose non-ferment 1 related protein kinase), and ABRE (ABA-responsive element binding protein)/*ABFs* (responsive element binding factor) genes [16]. The overexpression of *PYL* significantly enhances ABA sensitivity and drought tolerance in plants [17]. *CBL1* (Calcineurin B-like protein 1) participates in the ABA signaling pathway by regulating the MAPK signaling cascade, which affects the regulation of antioxidant defense and stomatal dynamics [18]. The MYB family is a key regulator in the drought stress response mechanism. As a positive regulatory transcription factor, the *ZmMYB3R* gene promotes higher ABA accumulation and enhances drought tolerance through an ABA-dependent pathway [19]. *HvMYB1* contributes to drought protection by serving as an intermediary for abscisic acid [20]. The sesame R2R3-MYB gene *SiMYB75* upregulates the expression levels of various stress marker genes via the ABA-dependent pathway and actively regulates responses to drought, salt, and osmotic stress through ABA-mediated pathways [21].

Excessive soil salinity can cause osmotic stress and ion toxicity in plants [22]. In response to salt stress, plants utilize ions for osmotic adjustment to minimize the harmful effects of Na^+^ ions. Na^+^/H^+^ antiporters, such as *NHX1* (Na^+^/H^+^ antiporters 1) and *SOS1* (salt overly sensitive 1), regulate Na^+^ sequestration in vacuoles and help to maintain osmotic balance [23,24]. *MYB42* phosphorylates and activates the Na^+^/H^+^ exchanger *SOS1* by regulating *SOS2* (salt overly sensitive 2), thereby promoting Na^+^ efflux to enhance plant salt tolerance [25]. The deletion of *AtMYB73* leads to the overexpression of the *SOS1* and *SOS3* (salt overly sensitive 3) genes, which enhances the salt tolerance of the mutant and improved survival rates [26]. Meanwhile, salt stress leads to the accumulation of ROS in plants. Superoxide dismutase (SOD) and catalase (CAT) serve as defense mechanisms to prevent oxidative damage and protect cells, thereby reducing ROS levels and maintaining redox homeostasis [27]. In *TaMYB86B* wheat high-expression lines exposed to salt stress, the transcription level of *TaSOD* is significantly up-regulated, improving salt tolerance by enhancing ROS scavenging and reducing membrane lipid peroxidation [28]. The overexpression of the *VhMYB15* gene in Arabidopsis increases the expression of *AtCAT1* and the accumulation of proline, thereby enhancing the ability to scavenge ROS [29]. In summary, the diversity of the studied species and their specific functions have laid a solid foundation for understanding the different roles of MYB transcription factors in abiotic stress responses. However, the verification of these abiotic-stress-responsive MYB proteins in blue honeysuckle remains limited.

Blue honeysuckle is an edible berry with high nutritional and economic value. In recent years, research and development on the utilization of its fruit’s nutritional properties have gradually increased. However, there are still relatively few studies on the cultivation mechanisms of blue honeysuckle, and its stress resistance mechanisms remain unclear. In a previous study, we examined the expression profiles of 56 R2R3-MYB genes in various tissues of blue honeysuckle under drought and salt stress conditions [30]. Among these, the *LcMYB90* gene was identified as having a significant response to both stress conditions. Consequently, we investigated the ectopic expression of the *LcMYB90* gene in transgenic tobacco and its dynamic expression in blue honeysuckle. The overexpression of the *LcMYB90* gene in transgenic plants significantly enhanced tolerance to drought and salt stress. Our results suggest that *LcMYB90* plays a crucial role in plant stress response and could serve as a candidate gene for improving drought and salt tolerance in future crop breeding programs.

## 2. Results

### 2.1. Bioinformatics Analysis of LcMYB90

**Figure 1:**
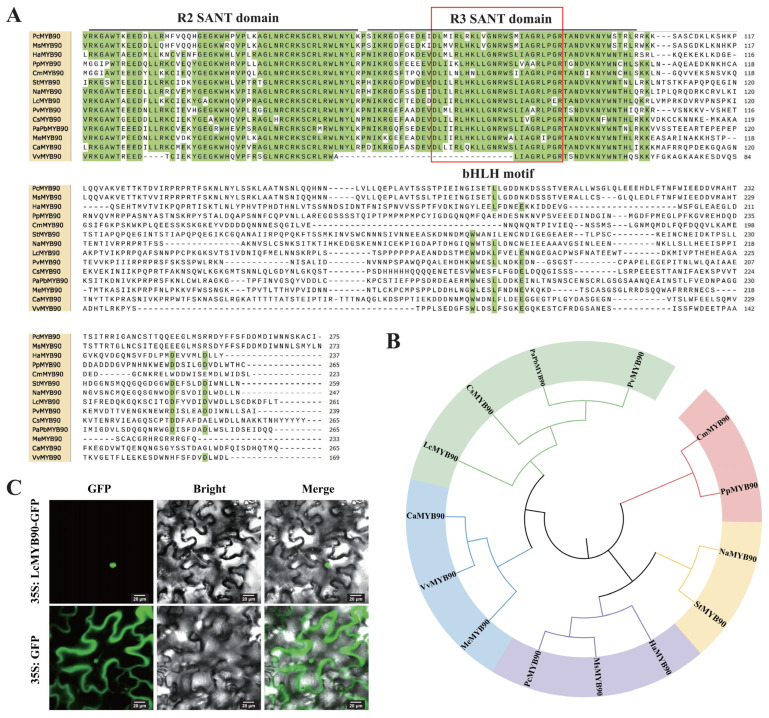
Sequence analysis and subcellular localization of the LcMYB90 protein. (**A**) Sequence alignment of LcMYB90 with other plant MYB90 proteins. Accession numbers include: PcMYB90 (*Pyrus communis*, XP_068316305.1), MsMYB90 (*Malus sylvestris*, XP_050107406.1), HaMYB90 (*Helianthus annuus*, XP_022033410.1), PpMYB90 (*Prunus persica*, XP_007205727.1), CmMYB90 (*Cucumis melo*, XP_008441809.2), StMYB90 (*Solanum tuberosum*, NP_001274774.1), NaMYB90 (*Nicotiana attenuata*, OIT20086.1), PvMYB90 (*Pistacia vera*, XP_031273872.1), CsMYB90 (*Citrus sinensis*, KAH9739068.1), PaPbMYB90 (*Populus alba* × *Populus* × *berolinensis*, KAJ6861420.1), MeMYB90 (*Manihot esculenta*, XP_043806607.1), CaMYB90 (*Coffea arabica*, XP_027073233.1), and VvMYB90 (*Vitis vinifera*, RVW22522.1). The red box represents a conserved motif interacting with bHLH. (**B**) Phylogenetic tree analysis of LcMYB90 and other plant MYB90 proteins. (**C**) Subcellular localization of LcMYB90 in tobacco leaf epidermal cells.

The cloned full-length cDNA of *LcMYB90* is 825 base pairs long and encodes a 274 amino acid protein. To investigate the evolutionary relationship between LcMYB90 and homologous proteins in other species, we performed multiple sequence alignments of MYB90 proteins from 13 different species, including *Prunus persica*, *Citrus sinensis*, and *Vitis vinifera*, etc. LcMYB90 contains a conserved R2 and R3 domain at the N-terminus, which exhibits the typical structural characteristics of the R2R3-MYB family (Figure 1A). The R2R3-MYB domain of LcMYB90 is highly conserved with its homologues. However, LcMYB90 shows some variability in other functional domains. Additionally, we identified a conserved motif [D/E]Lx2[R/K]x3Lx6Lx3R within the R3 domain of LcMYB90 that interacts with bHLH proteins. The presence of this motif suggests that LcMYB90 may bind to bHLH and fulfill a functional role. Phylogenetic analysis further demonstrated that LcMYB90 is most closely related to the MYB90 protein from *Citrus sinensis* (KAH9739068.1), indicating a strong genetic similarity and shared evolutionary history (Figure 1B).

### 2.2. LcMYB90 Is Localized in the Nucleus

Subcellular localization studies have determined the specific cellular location where the LcMYB90 protein functions. As shown in Figure 1C, under fluorescence confocal microscopy, the green fluorescent protein (GFP) control was broadly expressed throughout the cell, while the fluorescence of the LcMYB90-GFP fusion protein was confined to the nucleus, indicating that the LcMYB90 protein localizes to the cell nucleus.

### 2.3. Physiological Changes in Transgenic Tobacco Under Drought Stress 

**Figure 2:**
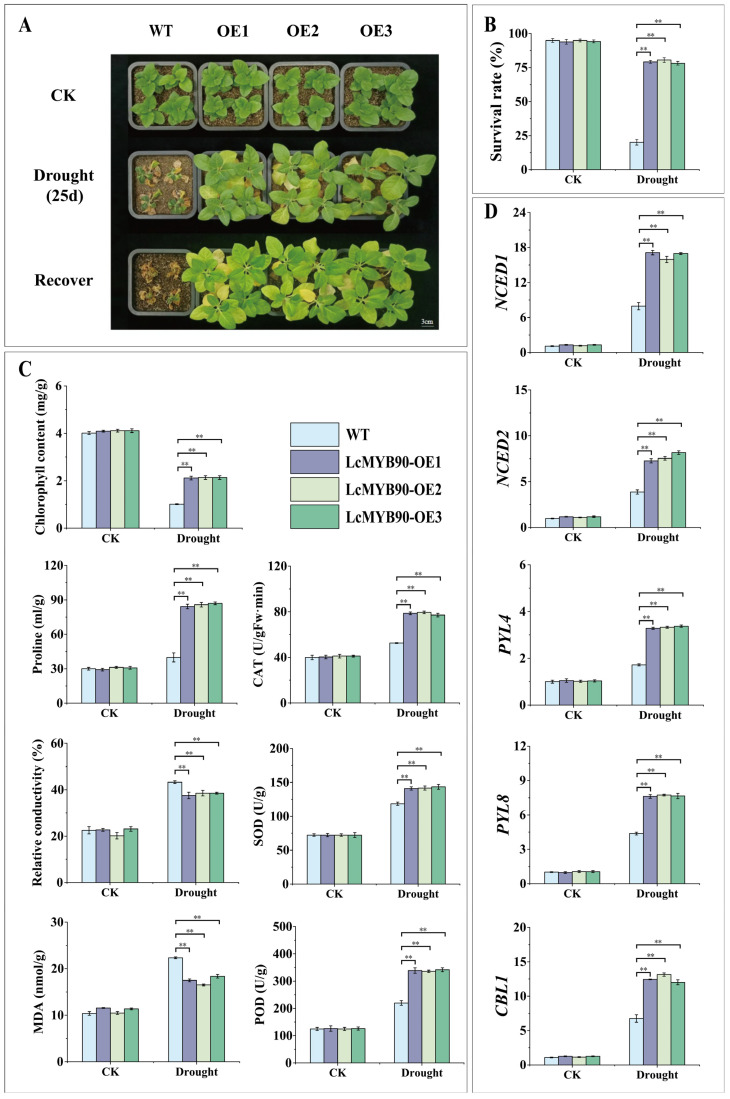
The figure demonstrates that *LcMYB90* overexpression enhances drought tolerance in tobacco. (**A**) Morphological alterations in tobacco due to drought stress. (**B**) Survival rate of tobacco after drought stress recovery. (**C**) Impact of the *LcMYB90* gene on physiological indicators of drought tolerance in tobacco. (**D**) Expression levels of drought-responsive genes in tobacco. Drought: natural drought. Asterisks denote significant differences from WT (** indicates *p* ≤ 0.01).

We produced transgenic tobacco plants with stable expression of the *LcMYB90* gene to study its role in drought stress (Figure S1A,C). Following 25 days of natural drought stress, both wild-type (WT) and transgenic LcMYB90 tobacco plants (OE1, OE2, and OE3) were rewatered. During this period, WT exhibited severe leaf wilting and dehydration, whereas OE1, OE2, and OE3 experienced significantly less damage (Figure 2A). Following 7 days of rewatering, the average survival rate of the transgenic tobacco was 85.32%, while the WT failed to recover, with a survival rate of only 20.18%, as most of the WT plants perished (Figure 2B).

We assessed malondialdehyde (MDA), proline, chlorophyll levels, relative electrical conductivity, and the enzymatic activities of CAT, SOD, and peroxidase (POD) in wild-type and *LcMYB90* transgenic tobacco lines (OE1, OE2, and OE3) under normal and drought stress conditions, as shown in Figure 2C. Prior to drought exposure, no significant differences in physiological parameters were observed among the different plants. Following drought treatment, all plants exhibited a reduction in chlorophyll content; however, the transgenic tobacco maintained a significantly higher level than the wild type. Concurrently, the activities of CAT, POD, and SOD, as well as proline levels, increased in all tobacco plants, with the transgenic tobacco exhibiting a more pronounced increase than the wild type. Under stress, both MDA levels and relative conductivity rose but were notably lower in the transgenic lines than in the wild type. These findings indicate that *LcMYB90* potentially improves drought resistance in transgenic tobacco.

### 2.4. Overexpression of LcMYB90 Increases the Expression of Drought-Related Genes in Transgenic Tobacco

MYB transcription factors modulate downstream gene expression in response to drought stress and are involved in the ABA signaling pathway, resulting in morphological and physiological changes that improve drought resistance in plants. Drought stress induces the expression of several related genes, including *PYL4* (abscisic acid receptor *PYL4*), *PYL8* (abscisic acid receptor *PYL8*), *NCED1* (9-cis-epoxy carotenoid dioxygenase 1), *NCED2* (9-cis-epoxy carotenoid dioxygenase 2), and *CBL1* (Figure 2D). On the 25th day of drought stress, gene expression levels increased in both the wild-type and the transgenic *LcMYB90* tobacco. The expression levels in the wild-type lines were significantly lower compared to the *LcMYB90*-overexpressing transgenic lines (OE1, OE2, and OE3). The *LcMYB90* transcription factor enhances plant drought resistance by upregulating *NCED1*, *NCED2*, *PYL4*, *PYL8*, and *CBL1* expression.

### 2.5. Physiological Changes in Transgenic Tobacco Under Salt Stress

**Figure 3:**
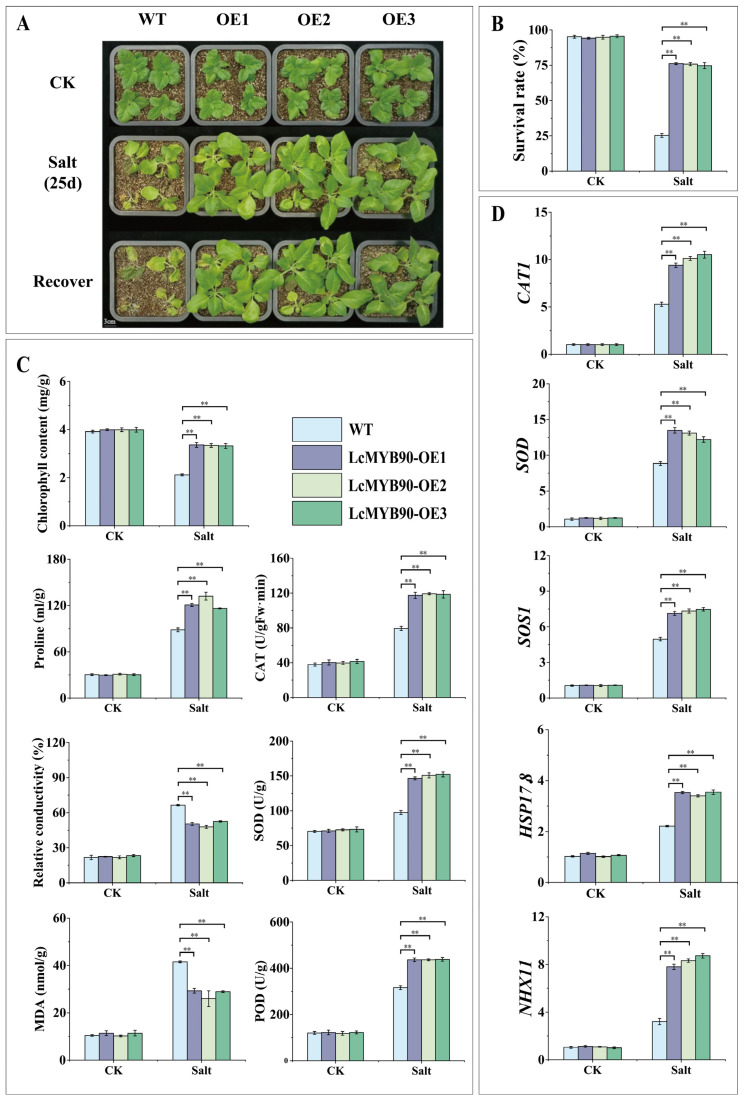
Overexpression of *LcMYB90* can improve salt tolerance in tobacco. (**A**) Morphological alterations in tobacco due to salt stress. (**B**) Survival rate of tobacco after salt stress recovery. (**C**) Impact of the *LcMYB90* gene on physiological indicators of salt tolerance in tobacco. (**D**) Expression levels of genes associated with salt stress in tobacco. Salt: Watering with 200 mM sodium chloride solution. Asterisks denote significant differences from WT (** indicates *p* ≤ 0.01).

After 14 days of irrigation with 200 mM sodium chloride solution for salt stress treatment, the wild-type tobacco (WT) and transgenic *LcMYB90* lines (OE1, OE2, and OE3) were returned to a normal growth environment. The salt treatment caused varying degrees of damage across all lines, with the wild-type tobacco exhibiting pronounced signs of wilting and dehydration (Figure 3A). After 7 days of recovery, the wild-type tobacco showed limited recovery, with a survival rate of only 25.11%. In contrast, the transgenic lines demonstrated high resilience, achieving an average survival rate of 75.75% (Figure 3B).

Before the salt stress treatment, physiological indexes showed no significant differences between the wild-type and transgenic tobacco (Figure 3C). Under salt stress, both the wild-type and transgenic tobacco exhibited increased activities of CAT, POD, and SOD, as well as elevated proline content, with the transgenic tobacco showing a more significant enhancement. Concurrently, salt stress led to a reduction in chlorophyll content across all tobacco lines, however, this reduction was less severe in the transgenic plants. Under high salt stress, both MDA content and relative conductivity increased, with a more pronounced rise in the wild-type than in transgenic tobacco, indicating that *LcMYB90* enhances salt tolerance in transgenic tobacco.

### 2.6. LcMYB90 Overexpression Enhances Salt-Related Gene Expression in Transgenic Tobacco

This study evaluated the downstream expression levels of several key MYB transcription factor genes under high salt concentrations (Figure 3D). After 14 days of salt stress, all plant samples showed significantly increased expression levels of *NHX1*, *SOD*, *CAT1*, *SOS1*, and small heat shock protein 17.8 (*HSP17.8*). The expression levels of these genes were significantly elevated in the *LcMYB90*-overexpressing lines compared to the wild type. The *LcMYB90* transcription factor appears to upregulate *NHX1*, *SOD*, *CAT1*, *SOS1*, and *HSP17.8*, which in turn boosts plant tolerance to salt stress.

### 2.7. Reaction of Blue Honeysuckle with Dynamic Expression of LcMYB90 to Drought Conditions

**Figure 4:**
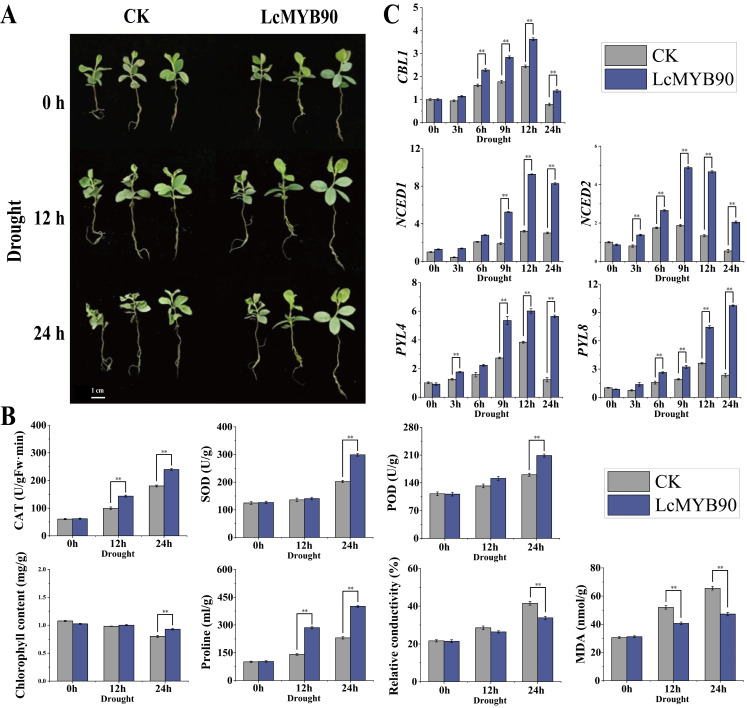
Overexpression of *LcMYB90* can improve drought tolerance in blue honeysuckle. (**A**) Morphological alterations in blue honeysuckle due to drought stress. (**B**) Effects of the *LcMYB90* gene on drought tolerance physiological indicators in blue honeysuckle. (**C**) Expression levels of genes responsive to drought in blue honeysuckle. Drought: 20% PEG600 Hoagland solution. Asterisks denote significant differences from WT (** indicates *p* ≤ 0.01).

By transient genetic transformation, we obtained blue honeysuckle seedlings overexpressing the *LcMYB90* gene, with expression levels more than three times higher than those found in the wild type (Figure S1D). To examine the *LcMYB90* gene’s role in blue honeysuckle’s drought stress response, both wild-type and *LcMYB90*-overexpressing seedlings were subjected to drought using a 20% PEG6000 solution. The morphological changes were evaluated at 0, 12, and 24 h after treatment. As depicted in Figure 4A, no notable phenotypic differences were observed among the lines at the 0 h mark. However, after 12 h treatment, the wild-type blue honeysuckle displayed visible signs of obvious water loss and wilting, while the *LcMYB90*-overexpressing seedlings showed no significant morphological changes. After 24 h of drought treatment, the *LcMYB90*-overexpressing seedlings experienced mild dehydration and wilting, in contrast to the wild-type plants, which exhibited severe dehydration, wilting, and, in some cases, mortality.

Within 24 h of drought stress, the CAT activity and proline levels in blue honeysuckle seedlings rose (Figure 4B). The *LcMYB90*-overexpressing plants exhibited more pronounced effects than the wild type. After 24 h of drought stress, POD and SOD activities increased significantly in all lines, with *LcMYB90*-overexpressing plants exhibiting notably higher activity than the wild type. Furthermore, stress led to increased MDA levels and relative electrical conductivity, with the wild-type plants exhibiting a greater rise than the *LcMYB90*-overexpressing lines. The *LcMYB90* gene enhances drought tolerance in blue honeysuckle.

### 2.8. Expression of Genes Related to Drought in Transiently Transformed LcMYB90 Blue Honeysuckle

We investigated the expression levels of drought-related genes in wild-type and *LcMYB90*-overexpressing blue honeysuckle seedlings under drought stress. In the *LcMYB90*-overexpressing blue honeysuckle seedlings, the expression levels of the key genes *NCED1*, *NCED2*, *PYL4*, *PYL8*, and *CBL1* progressively increased at 0, 3, 6, and 9 h following drought treatment (Figure 4C). *NCED1* expression peaked at 12 h of drought stress, showing an 8.2-fold increase in the *LcMYB90*-overexpressing plants compared to normal levels (at 0 h), which is about three times higher than that observed in the wild-type plants at the same time.

The expression levels of *NCED2*, *PYL4*, and *CBL1* initially rose, reaching their highest at 9 and 12 h of stress, respectively, before decreasing. Notably, the transgenic plants showed higher peak expression levels than the wild type. In the wild-type plants, *PYL8* expression initially increased and then decreased, whereas the *LcMYB90*-overexpressing plants showed a continuous increase, peaking at 24 h. At this peak, *PYL8* expression in the transgenic plants was 10.5 times higher than that observed at 0 h and approximately 4.1 times greater than that found in the wild-type plants. The result suggests that *LcMYB90* positively influences the expression of these genes, contributing to improved drought resistance in blue honeysuckle.

### 2.9. Salt Stress Response of Transiently Transformed LcMYB90 in Blue Honeysuckle

**Figure 5:**
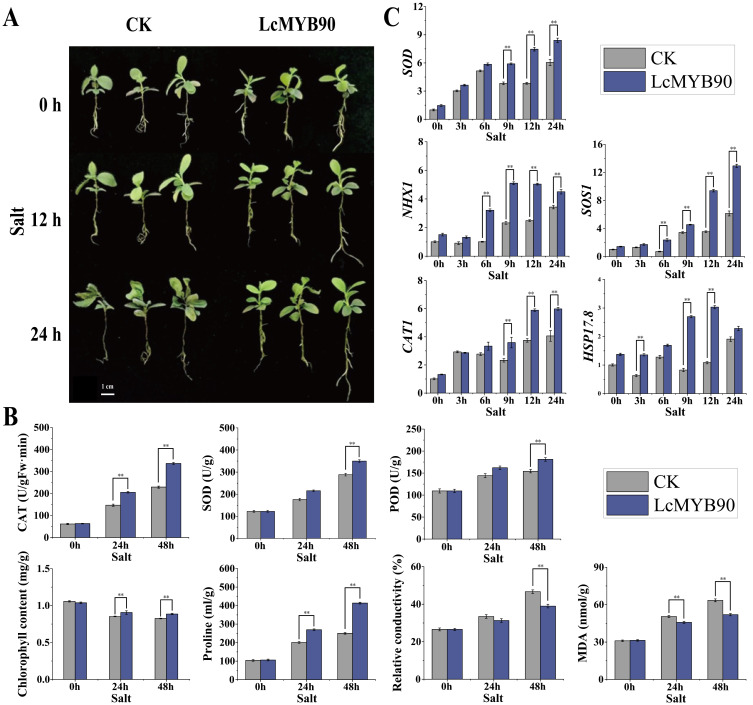
The figure demonstrates that *LcMYB90* overexpression enhances salt tolerance in blue honeysuckle. (**A**) Morphological alterations in blue honeysuckle due to salt stress. (**B**) Effects of the *LcMYB90* gene on salt tolerance physiological indicators in blue honeysuckle. (**C**) Expression levels of salt-stress-related genes in blue honeysuckle. Salt: Hoagland solution containing 200 mM sodium chloride. Asterisks denote significant differences from WT (** indicates *p* ≤ 0.01).

Under normal growth conditions, transgenic plants showed no significant morphological differences compared with wild-type plants. To explore the function of the *LcMYB90* gene in blue honeysuckle under salt stress, we treated both the wild-type and *LcMYB90*-overexpressing seedlings with a 200 mM sodium chloride solution. After 48 h of salt stress treatment, the wild-type blue honeysuckle seedlings exhibited significant wilting, with some plants dying (Figure 5A). In contrast, blue honeysuckle seedlings overexpressing *LcMYB90* displayed only mild dehydration and wilting. The activities of CAT, POD, and SOD, as well as the proline levels, in the two plant types continued to increase within 24 and 48 h of salt stress (Figure 5B). The increase was significantly more pronounced in the *LcMYB90*-overexpressing plants compared to the wild type. Similarly, the levels of MDA and relative electrical conductivity also increased, while a significant reduction in chlorophyll content was observed. However, for MDA and relative conductivity, the wild-type plants exhibited a greater increase. These findings suggest that the *LcMYB90*-overexpressing blue honeysuckle possesses improved tolerance to salt stress.

### 2.10. Expression of Salt-Responsive Genes in Transiently Transformed LcMYB90 Blue Honeysuckle

The qRT-PCR indicated an elevated expression of *NHX1*, *SOD*, *CAT1*, *SOS1*, and *HSP17.8* genes in the *LcMYB90*-overexpressing blue honeysuckle seedlings under salt stress. Their expression levels were significantly up-regulated compared to the pre-treatment levels and were notably higher than those found in the wild type. *NHX1* showed an expression peak at 18 h of salt treatment in the *LcMYB90*-overexpressing lines, approximately 3.4 times higher than that observed in the unprocessed sample and 2.2 times higher than that seen in the in the wild type. *SOD*, *CAT1*, and *SOS1* genes in the *LcMYB90*-overexpressing lines exhibited a sustained increase in expression, reaching peak levels at 48 h of salt treatment.

Following 48 h of salt stress, the expression levels of *SOD*, *CAT1*, and *SOS1* increased by 5.7-, 4.6-, and 9.3-fold, respectively, compared to the untreated group, while the wild type exhibited increases of 1.4-, 1.5-, and 2.1-fold, respectively. *HSP17.8* expression in the overexpression line was consistently elevated compared to the wild type, showing a significant increase at 12 h of salt treatment, reaching approximately 2.2 times that of the untreated group and 2.8 times that of the wild type.

## 3. Discussion

### 3.1. Role of LcMYB90 in Drought Stress Response

**Figure 6:**
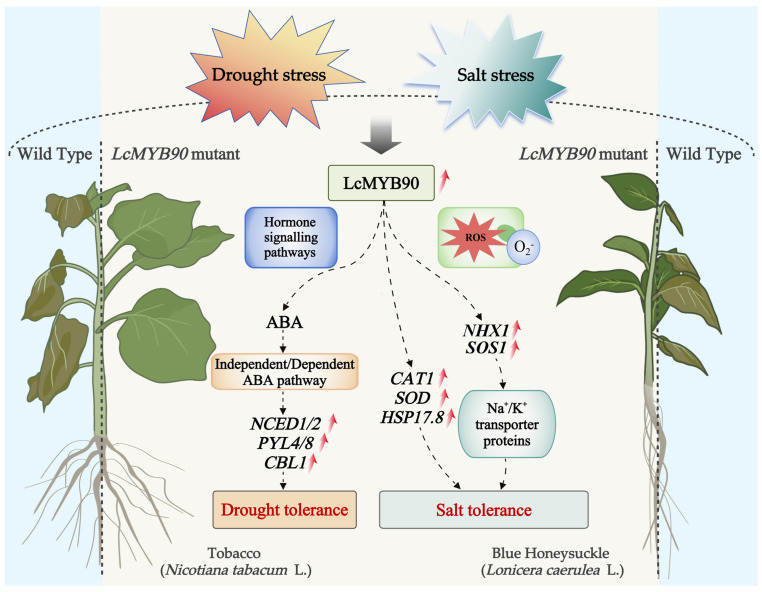
A model of possible mechanisms of *LcMYB90* in response to drought and salt stress. Gray, red and dotted arrows represent stress treatment, gene expression trends, and indirect regulatory relationships, respectively.

This study found that transgenic tobacco and blue honeysuckle plants overexpressing *LcMYB90* showed less leaf and root damage and significantly improved survival rates under drought stress compared to wild-type plants. This indicates an enhancement in drought tolerance. To elucidate the role of the *LcMYB90* gene under drought stress, we further investigated its impact on plant physiological parameters and molecular mechanisms and mapped the possible mechanism model of *LcMYB90* in response to drought and salt stress (Figure 6).

#### 3.1.1. Changes in Plant Physiological and Biochemical Indices Under Drought Conditions

Metabolic disorders caused by drought can increase ROS, which subsequently affect cellular redox regulation. Redox regulation and antioxidant systems are pivotal in the recovery efficiency of plant normal function and plant traits, significantly impacting drought resistance. Enhanced SOD activity facilitates the conversion of superoxide anions into hydrogen peroxide, addressing the ROS surge due to water scarcity. However, POD and CAT change synergistically with SOD, and the activity increases significantly at the initial stage of drought stress, which is involved in further scavenging hydrogen peroxide. This synergistic effect helps to maintain intracellular redox homeostasis. Drought stress significantly reduces plant chlorophyll content, affects photosynthetic efficiency, and increases malondialdehyde content, causing severe oxidative damage to cell membranes. Additionally, drought stress leads to an increase in plant cell membrane permeability and electrolyte leakage, thereby raising relative conductivity. In this process, the proline content in plants increases significantly, serving as a protective response.

Research indicates that birch *BplMYB46* enhances drought resistance by upregulating genes like *SOD*, *POD*, and *P5CS* (Pyrroline-5-carboxylate synthase), which boost ROS scavenging and proline accumulation [31]. Wheat *TaMYB22* responds to drought stress by participating in the ABA signaling pathway, enhancing drought resistance in plants by increasing antioxidant enzyme activity and accumulating osmotic regulatory substances [32]. This study showed that *LcMYB90* overexpression in tobacco and blue honeysuckle seedlings under drought stress significantly enhances POD, CAT, SOD enzyme activities, as well as proline content, compared to the wild type, while reducing the drought-induced rapid increase in MDA content and relative conductivity. These changes in physiological indices indicate that the *LcMYB90* gene can promote the rapid clearance of ROS in plants, reduce plant damage, and further demonstrate its role in enhancing plant drought resistance.

#### 3.1.2. Regulation of Drought-Stress-Related Genes by *LcMYB90*

NCED is a crucial enzyme that limits the rate of ABA biosynthesis in plants, aiding in the reduction in plant damage during drought conditions. Drought stress induces the expression of the *NCED1* gene in kidney beans, and the overexpression of *NCED1* can promote ABA accumulation, enhancing drought resistance in transgenic plants [33]. Similarly, *NCED2* expression in plant roots and leaves significantly rises under drought stress, facilitating ABA accumulation and enhancing drought resistance [34]. The *PYL* gene family functions as abscisic acid receptors, engaging in the ABA signaling pathway to respond to drought stress. Research indicates that *PYL8* interacts with transcription factors *MYB77*, *MYB44*, and *MYB73*, playing a role in ABA signaling and enhancing drought stress responses [35]. In tomatoes, the *PYL4* gene was significantly upregulated after exogenous treatment with the ABA hormone [16]. The CBL family of calcium-dependent protein kinases in plants serves as a unique group of calcium sensors, with *CBL1* being notably upregulated in response to diverse stress signals. Research indicates that the overexpression of *CBL1* in plants enhances drought stress tolerance, with *CBL1* serving as a positive regulator of the drought response [36]. In Arabidopsis thaliana transformed with the R2R3-MYB family gene *PtrSSR1*, the expression levels of ABA-related genes *NCED3*, *ABI1*, and *CBL1* were significantly upregulated, leading to enhanced salt tolerance in transgenic Arabidopsis [37]. This study demonstrates that *LcMYB90* overexpression significantly elevates the expression of *PYL4*, *PYL8*, *NCED1*, *NCED2*, and *CBL1* genes in tobacco and blue honeysuckle compared to the wild type. This suggests that *LcMYB90* positively regulates these genes, thereby modulating the ABA signal transduction pathway and improving plant drought resistance.

### 3.2. Involvement of LcMYB90 in Response to Salt Stress

This study examined transgenic tobacco and blue honeysuckle plants overexpressing *LcMYB90* under salt stress, analyzing their physiological and biochemical indices and the expression of salt-resistant genes. The transgenic *LcMYB90* lines exhibited significantly greater salt tolerance than the wild type.

#### 3.2.1. Changes in Plant Physiological and Biochemical Indices Under Salt Stress

To adapt to salt stress, plants have developed complex physiological and biochemical regulatory mechanisms to cope with adverse environmental conditions. Under salt stress, plants generate osmotic regulators like proline, betaine, and soluble sugars to sustain cell turgor and metabolism. Moreover, plants have an advanced antioxidant system that includes crucial enzymes like SOD, CAT, POD, ascorbate peroxidase, and glutathione peroxidase, which generally show a significant rise in activity when faced with salt stress. In our study, we found that the activity levels of CAT, POD, and SOD, as well as the proline content and relative conductivity of plants overexpressing *LcMYB90*, increased to varying degrees in response to salt stress. These results align with earlier research. This indicates that *LcMYB90* can regulate these indicators, swiftly remove ROS in plants, reduce plant damage, and thereby enhance plant salt tolerance.

#### 3.2.2. Regulation of Salt-Stress-Related Genes by *LcMYB90*

Na^+^/H^+^ exchangers *NHX1* and *SOS1* play important roles in plant salt tolerance by regulating Na^+^/H^+^ reverse transport proteins. Overexpressing the *PeMYB2* gene in bamboo improves salt tolerance in transgenic plants, with elevated expression levels of key salt stress response genes such as *NXH1*, *SOS1*, *RD29A* (responsive to desiccation 29A), and *COR15A* (cold-regulated 15A) compared to wild-type plants [38]. Research indicated a significant increase in *SOD* and *CAT* expression levels in grafted cucumber leaves under salt stress [39]. The expression levels of heat shock protein genes *Hsp26.3*, *Hsp17.8*, and *Hsp101* increased significantly in wheat leaves after salt stress treatment, with increased proline content and decreased chlorophyll content [35]. This study revealed that *LcMYB90*-overexpressing plants exhibited significantly elevated expression levels of *NHX1*, *SOD*, *CAT1*, *SOS1*, and *HSP17.8* genes compared to wild-type plants following salt stress. This suggests that *LcMYB90* positively regulates these genes and enhances plant resistance to salt stress.

## 4. Materials and Methods

### 4.1. Plant Materials

The ten-year-old blue honeysuckle cultivar ‘Lanjingling’ produced by Northeast Agricultural University in Harbin, China, is the plant material used in this study. Mature seeds were harvested from fully developed, ripe fruits and stored in a humid environment. Germination took place at a temperature of 21 ± 2 °C, accompanied by a 12 h light and 12 h dark photoperiod. The growing medium, a sterilized blend of peat soil, vermiculite, and perlite in equal proportions (1:1:1), underwent sterilization through high temperature and pressure. Post-germination, the seeds were transferred to seedling trays and cultivated for 40 days under controlled conditions, as follows: 25 ± 2 °C temperature, 16 h light/8 h dark photoperiod, and 60–70% relative humidity.

### 4.2. Phylogenetic Analysis

The nucleotide sequence of the *LcMYB90* gene was submitted to genebank and the GenBank accession number (BankIt2912465 BSeq#1 PQ867620) was obtained. Blast analysis of the LcMYB90 protein sequence was conducted using the NCBI database (http://www.ncbi.nlm.nih.gov/ accessed on 5 March 2023). A total of 13 MYB90 protein sequences similar to LcMYB90 were identified, including PcMYB90 (*Pyrus communis*, XP_068316305.1), MsMYB90 (*Malus sylvestris*, XP_050107406.1), HaMYB90 (*Helianthus annuus*, XP_022033410.1), PpMYB90 (*Prunus persica*, XP_007205727.1), CmMYB90 (*Cucumis melo*, XP_008441809.2), StMYB90 (*Solanum tuberosum*, NP_001274774.1), NaMYB90 (*Nicotiana attenuata*, OIT20086.1), PvMYB90 (*Pistacia vera*, XP_031273872.1), CsMYB90 (*Citrus sinensis*, KAH9739068.1), PaPbMYB90 (*Populus alba* × *Populus* × *berolinensis*, KAJ6861420.1), MeMYB90 (*Manihot esculenta*, XP_043806607.1), CaMYB90 (*Coffea arabica*, XP_027073233.1), and VvMYB90 (*Vitis vinifera*, RVW22522.1). MEGA 11 software was utilized for multiple sequence alignment and phylogenetic tree construction.

### 4.3. Genetic Transformation in Tobacco of LcMYB90

Total RNA was extracted from mature blue honeysuckle fruits using the E.Z.N.A. Plant RNA Kit (Omega Bio-Tek, Guangzhou, China) and purified with RNase-Free DNase I. cDNA synthesis was performed using the TOYOBO reverse transcription kit (TOYOBO, Shanghai, China). A full-length open reading frame for *LcMYB90* was PCR-amplified with primers *LcMYB90*_F_BamH I (CGCGGATCCATGGAGAGTAATAGTAAAGGTAGTAG) and *LcMYB90*_R_Xba I (GCTCTAGAAGTCAAGAAGTCTTTATCACAAC) (introduced BamH I and Xba I sites are underlined). The synthesized cDNA underwent amplification, followed by purification of the PCR products. These fragments were digested with BamHI and XbaI enzymes and then ligated into the pCAMBIA1300_GFP vector using T4 ligase. Primer sequences are provided in Table S1. Ligation products were introduced into Escherichia coli (DH5α)-competent cells through heat shock, and correctly sequenced plasmids were transferred into the *Agrobacterium tumefaciens* strain GV3101 for tobacco transformation using the *Agrobacterium*-mediated method [16].

Vigorous and morphologically intact transgenic tobacco calli were cultured in an MS medium with 0.5 mg/L 6-BA and 0.05 mg/L NAA. The calli were transferred to an MS rooting medium with 1.5 mg/L IBA to induce root formation. Uniform and healthy seedlings were selected and transplanted into seedling pots (18 cm × 18 cm) containing a soil mixture of peat soil, vermiculite, and perlite in a 1:1:1 ratio. The plants were grown under controlled conditions on tissue culture shelves at 25 ± 2 °C with a 16 h light/8 h dark cycle for subsequent experiments.

### 4.4. Temporary Transformation of Blue Honeysuckle

The *Agrobacterium* GV3101 culture was cultivated to an OD600 of 0.5 and then centrifuged at 6000 rpm for 10 min to collect the bacterial pellet. The bacterial precipitate was resuspended in 1/2 MS solution at a volume five times greater and incubated overnight at 28 °C with shaking at 200 rpm. Hydroponically grown blue honeysuckle seedlings were submerged in this bacterial liquid overnight, which was enhanced with 100 mM acetosyringone (AS) to a final concentration (100 mL bacterial liquid + 150 μM AS). The seedlings were agitated at 28 °C and 100 rpm for 5–6 h. Afterward, the seedlings were taken out, and excess liquid was blotted with sterile filter paper. Co-culture was continued in a dark environment for 48 h. The upstream primer was *LcMYB90*_F_BamH I, and the downstream primer was pCAMIBA1300-GFP-R (Table S1). After PCR, the success of transient transformation was verified based on the position of the bands in the gel electrophoresis (Figure S1B). The samples from three trees with similar plant sizes were selected together as a sample group, and three independent replicates were set for each experiment [30].

### 4.5. Drought Stress and Salt Treatment

Wild-type tobacco and *LcMYB90* transgenic tobacco plants with consistent growth potential were selected for a natural drought treatment by ceasing watering. The samples were collected at 0 days and 25 days for physiological index determination. The tobacco plants in the salt stress treatment group received daily irrigation of 200 mM sodium chloride solution, with samples being collected on days 0 and 14 post-treatment.

Drought stress on the blue honeysuckle seedlings was induced by transferring both normal and transiently transformed *LcMYB90* plants to a 20% PEG6000 Hoagland medium. Sampling occurred at 0, 3, 6, 9, 12, and 24 h following drought treatment. For salt stress treatment, the seedlings were exposed to a 200 mM sodium-chloride-enriched Hoagland nutrient solution. All transgenic blue honeysuckle seedlings were divided into three mixed sample groups for subsequent gene function verification experiments. The samples were collected at 0, 6, 12, 18, 24, and 48 h post-treatment.

### 4.6. Subcellular Localization

A modified red-shifted GFP existed in the pCAMBIA1300-*LcMYB90*-GFP vector. Transformation of the recombinant plasmid into tobacco epidermal cells by agrobacterium tumefaciens injection technology was conducted, with an empty 35S plasmid used as a control. DAPI staining was used as a nuclear marker. The transient expression of the *LcMYB90*-GFP fusion protein was detected under an TCS SP8 laser confocal microscope (Leica, Wetzlar, Germany).

### 4.7. Real-Time Quantitative PCR

Key stress-responsive genes were validated using quantitative real-time PCR (qRT-PCR). Specific quantitative primers were designed according to part of the gene sequence using Primer 6.0 software. The primer sequences of the *LcMYB90*, tobacco stress-related genes, and blue honeysuckle stress-related genes are listed in Tables S1, S2 and S3, respectively. The qRT-PCR protocol started with an initial 60 s procedure at 95 °C, followed by 40 cycles of 15 s at 95 °C, 20 s at 60 °C, and 15 s at 72 °C. A concluding extension was conducted at 72 °C for 4 min. The gene expression levels were quantified using the 2^−ΔΔCt^ method with β-Actin as the internal reference gene. Analyses were conducted using three biological replicates.

### 4.8. Measurement of Physiological Parameters

Chlorophyll solution absorbance was measured for each sample [40], and the chlorophyll content was determined using Wellburn’s formula [41]. POD and SOD activities were assessed with the Plant Detection Kit from Beijing Solarbio.

CAT activity [42] was determined using potassium permanganate titration. Leaves (0.2 g) were weighed and ground, followed by enzyme extraction in ultrapure water. The reaction mixture contained 1 mL of 0.1% guaiacol solution, 1 mL of 0.18% hydrogen peroxide solution, and 1 mL of enzyme solution. The absorbance of a 200 μL aliquot at 470 nm was measured using a microplate reader.

To determine the MDA content [43], 0.2 g of leaves was extracted in 3 mL of 10% trichloroacetic acid (TCA) and centrifuged at 10,000× *g* for 10 min. The supernatant (1 mL) was mixed with 1 mL of 0.5% thiobarbituric acid in 10% TCA and incubated in a 95 °C water bath for 30 min. After centrifugation at 10,000× *g* for 10 min, the absorbance of the supernatant was measured at 532 nm.

The proline content was determined [44]. Leaves (0.5 g) were extracted in 5 mL of 3% sulfosalicylic acid at 95 °C for 15 min. After centrifugation, 2 mL of the supernatant was transferred to a new test tube containing 2 mL of acetic acid and 2 mL of acidified ninhydrin reagent. Following incubation at 95 °C for 30 min, 5 mL of toluene was added to the test tube and mixed thoroughly to extract the red product. The absorbance of the toluene layer was measured at 532 nm.

The relative conductivity was measured using the pumping method [45]. The 0.1 g sample was weighed and placed in deionized water for 3 h after vacuum treatment, after which the conductivity of the extract was measured using a conductivity meter (R1). The sample was then heated in a boiling water bath for 30 min. After cooling to room temperature, the mixture was shaken well, and the conductivity of the extract was measured again (R2). This process was repeated three times. The relative conductivity was calculated as (R1/R2) × 100%.

### 4.9. Statistical Analysis

One-way analysis of variance (ANOVA) was performed using SPSS version 21.0. Standard deviations (SD) were calculated based on the mean values of three replicate experiments. Asterisks denote statistical significance: * for *p* ≤ 0.05 and ** for *p* ≤ 0.01.

## 5. Conclusions

In this study, a new MYB gene, named *LcMYB90*, was isolated from blue honeysuckle. According to the results of subcellular localization, the LcMYB90 protein was localized in the nucleus. Our findings indicate that the overexpression of the *LcMYB90* gene markedly improves drought and salt tolerance in tobacco and blue honeysuckle plants. This improvement is primarily accomplished by modulating MDA, proline, chlorophyll levels, and the activities of SOD, POD, and CAT to mitigate drought and salt stress damage. The *LcMYB90* gene enhances the expression of crucial drought stress genes (*NCED1*, *NCED2*, *PYL4*, *PYL8*, and *CBL1*) and key salt stress genes (*NHX1*, *SOD*, *CAT1*, *SOS1*, and *HSP17.8*). These results suggest that the *LcMYB90* gene is a stress-responsive transcription factor that actively regulates drought and salt stress resistance. It holds potential as a candidate gene for molecular breeding of salt- and drought-tolerant crop varieties. Subsequently, further verification by gene silencing is needed to confirm the pivotal role of *LcMYB90* in abiotic stress tolerance.

## Data Availability

The original data in the present study are available from the corresponding authors.

## Supplementary Materials

The following supporting information can be downloaded at: https://www.mdpi.com/article/10.3390/ijms26073124/s1.

## Abbreviations

SOD: superoxide dismutase; CAT: catalase; POD: peroxidase; MDA: malondialdehyde; GFP: green fluorescent protein; ABA: abscisic acid; ROS: reactive oxygen species; *NHX1*: Na^+^/H^+^ antiporters 1; *SOS1*: salt overly sensitive 1; *SOS2*: salt overly sensitive 2; *SOS3*: salt overly sensitive 3; *SOD*: superoxide dismutase; *CAT1*: catalase 1; *NCED1*: 9-cis-epoxycarotenoid dioxygenase 1; *NCED2*: 9-cis-epoxycarotenoid dioxygenase 2; *PYL4*: pyrabactin resistance-like 4; *PYL8*: pyrabactin resistance-like 8; *CBL1*: Calcineurin B-like protein 1; *PP2C*: protein phosphatase 2C; *SnRK*: Sucrose non-ferment 1 related protein kinase; *ABRE*: ABA-responsive element binding protein; *ABFs*: responsive element binding factor; *P5CS*: Pyrroline-5-carboxylate synthase.
