# Supplementary material for: Overexpression of LcMYB90 Transcription Factor Enhances Drought and Salt Tolerance in Blue Honeysuckle (Lonicera caerulea L.) and Tobacco (Nicotiana tabacum L.)
Source: Int J Mol Sci. 2025 Mar 28;26(7):3124. doi: 10.3390/ijms26073124 (PMC11988839; doi:10.3390/ijms26073124)
Supplement: Supplementary file 1 [file ijms-26-03124-s001.zip › Table S1.pdf]

Table S1. Primers of *LcMYB90* used in this study

Primer Name	Primer sequence (5'→3')
*LcMYB*90_F_*Bam*H I	CGCGGATCCATGGAGAGTAATAGTAAAGGTAGTAG
*LcMYB*90_R_*Xba* I	GCTCTAGAAGTCAAGAAGTCTTTATCACAAC
pCABIA1300_GFP_F	ACGCACAATCCCACTATCCTTC
pCABIA1300_GFP_R	CGTCGCCGTCCAGCTCGACCAG
q-*LcMYB90*-F	AGACCTCAAGCCTTCTCCAATA
q-*LcMYB90*-R	GTGGTGGTGGTGATGTTGATAA
