# Supplementary material for: Overexpression of LcMYB90 Transcription Factor Enhances Drought and Salt Tolerance in Blue Honeysuckle (Lonicera caerulea L.) and Tobacco (Nicotiana tabacum L.)
Source: Int J Mol Sci. 2025 Mar 28;26(7):3124. doi: 10.3390/ijms26073124 (PMC11988839; doi:10.3390/ijms26073124)
Supplement: Supplementary file 1 [file ijms-26-03124-s001.zip › Table S2.pdf]

Table S2. qRT-PCR primer sequences for key genes of *Nicotiana tabacum* stresses

Primer Name	Primer sequence (5'→3')
PYL4-F	AGAATCAACCCAAGCACC
PYL4-R	CAGGGACAGAAACGGAAA
NCED1-F	TCACTCCAAGCTCCTCCA
NCED1-R	CACTTTCCACGGCATCTA
NCED2-F	TAATTCCCGACCAGCAAG
NCED2-R	TTCCCAAGCATTCCATAA
PYL8-F	ACGGAAGACCTGGGACAC
PYL8-R	AAGCCGCTCAGAAACATC
CBL1-F	ATTAGCGGTTCGGTAGTTG
CBL1-R	TTGTGAGGCATTTGGGTG
NHX1-F	TCTGCTGGAGGAGAACCG
NHX1-R	ACCCTGCATTAAATATGATTGG
HSP17.8-F	CAACGCTCGAATTGATTG
HSP17.8-R	CATACGGTGCCATTTGTC
CAT1-F	ATACAAGTACCGTCCGTCAA
CAT1-R	CTCGGGCATGAACAACAC
SOD-F	TCCTAATGCTGTAAACCCTC
SOD-R	CCTAGAACTCACCGCCTC
SOS1-F	AGTTCCTTACTCTGTCGCTTTG
SOS1-R	TGCCCAGATACGAATACCAT
Actin7-q-F	TGCTGATCGTATGAGCAAGG
Actin7-q-R	ATCCTCCGATCCAGACACTG
