# Supplementary material for: Overexpression of LcMYB90 Transcription Factor Enhances Drought and Salt Tolerance in Blue Honeysuckle (Lonicera caerulea L.) and Tobacco (Nicotiana tabacum L.)
Source: Int J Mol Sci. 2025 Mar 28;26(7):3124. doi: 10.3390/ijms26073124 (PMC11988839; doi:10.3390/ijms26073124)
Supplement: Supplementary file 1 [file ijms-26-03124-s001.zip › Table S3.pdf]

Table S3. qRT-PCR primer sequences for key genes of blue honeysuckle stresses

Primer Name	Primer sequence (5'→3')
PYL4-F	ATCTCCGCCCCCCTTTCC
PYL4-R	GTGCCGACGCTCCCTTCC
NCED1-F	TTCACCGAAACCGAAAGA
NCED1-R	GATCAAGGAGCCCCACTA
NCED2-F	CTACAAAGAGCCGCATCA
NCED2-R	GGATACGGGTAGGTTATGTTT
PYL8-F	GTAAGCAGGTGCGTTGTG
PYL8-R	CTGGTAGTGGCAGGAAGG
CBL1-F	TGATTGACTTTGGCGACTT
CBL1-R	TTGCCGCTCAATAAATCC
NHX1-F	TGGCACAATGTTACCGAAAG
NHX1-R	AACGCATCCATACCCACA
HSP17.8-F	ATGCAATTCAAGCCAAGC
HSP17.8-R	ATCGCATTAGAGGTCAGGTT
CAT1-F	TTGCAGACATTTTAGACC
CAT1-R	ATGCAAATAAGCTAAGGA
SOD-F	GGGCTGGGCTGTTGATAC
SOD-R	ATGCCAAGCAGAGGAACCA
SOS1-F	GATGGCATACGGCTTTGG
SOS1-R	CACCAGGCCCAGCAAGTA
β-Actin-F	ACCTGCTGACGAGTGCCGATAC
β-Actin-R	TCACCCTTGAAACATCAGGAGACCA
